# Support for Community School Personnel Working with Pediatric Cancer Patients: A Quality Improvement Initiative

**DOI:** 10.5334/cie.36

**Published:** 2022-01-20

**Authors:** Sarah Klein, Nicole Byford, Susan Ellison, Niki Jurbergs

**Affiliations:** 1 St. Jude Children’s Research Hospital, US

**Keywords:** school reentry, pediatric oncology, education, psychosocial support, school personnel

## Abstract

Improved therapies and increased survival rates are sending more pediatric cancer patients and survivors back to their classrooms; however, most community school personnel lack training or experience in working with these students. The aim of this quality improvement project was twofold: (a) to evaluate community school personnel’s perceptions of their preparedness to work with patients and childhood cancer survivors who have reentered the classroom; and (b) to standardize school reentry supports to improve community school personnel preparedness. Twenty community school personnel, prekindergarten through 12-grade teachers, guidance counselors, and administrators, who had previously worked with a pediatric cancer patient were surveyed regarding their experiences with the patient’s school reentry. Responses were coded and analyzed, and a thematic map was created. School personnel reported concerns related to student functioning, such as academic readiness, cognitive impacts of treatment, social-emotional adjustment, physical ability to participate in school, and medical fragility. They also reported concerns related to their own ability to accommodate the student’s needs. These results were used to design educational guides for community personnel consisting of information and resources to support them in managing the unique academic, social-emotional, physical, and medical needs of pediatric cancer patients and survivors in the classroom.

In 2021, it is estimated that 15,590 children and adolescents will be diagnosed with cancer in the United States, and 13,810 will survive their disease (Siegel et al., 2021). Students diagnosed with cancer are often absent from school due to treatment and treatment-related side effects. Students who are chronically absent from school tend to have low academic performance and high drop-out rates. Further, lengthy treatment protocols and intense treatment regimens exacerbate the issues that accompany prolonged absenteeism (Prevatt et al., 2000).

Missed instruction paired with the acute cancer and treatment-related effects (e.g., pain, fatigue, decreased immune functioning, changes in physical appearance and physical functioning) and late cognitive effects (e.g., reduced attention, working memory, and processing speed) can make returning to the classroom difficult for both patients and the school teams who support them (Harman et al., 2019; Jacola et al., 2016; Knight et al., 2014; Reddick et al., 2014; Winter et al., 2014).

School reentry support is a widely accepted psychosocial standard of care for children with cancer and should include the provision of information to school personnel about the patient’s diagnosis, treatment, impact on learning, as well as recommendations for how to support the student in the classroom (Thompson et al., 2015). Guidelines developed by Wiener and colleagues (2020) describe essential elements to meet this standard, including providing educational resources for teachers on academic, physical, social, and emotional support for patients over the course of treatment. Further, the Association of Pediatric Hematology Oncology Education Specialists (APHOES) (2015) recommends “clear, on-going communication between [the hospital team], child, parents, and teachers,” with meetings of stakeholders being held at the time of school reentry, the beginning of new academic years, and during the transition to a new school (6). Finally, the International Society of Pediatric Oncology (SIOP) has also documented the need for school reentry programming and open communication between the hospital team and community school personnel, including creating a manual for teachers (Masera et al., 1995).

The process of re-entering school after diagnosis and treatment can be challenging for the patient, parents, peers, and school personnel, and the need for strategic support is evident.

Well-developed training should provide information, guidance, and support to school personnel responsible for meeting the academic, social, and emotional needs of pediatric cancer patients and survivors in their classrooms.

Dedicated curriculum to prepare preservice teachers to support the needs of students with chronic health conditions, including pediatric cancer, is limited. College courses focusing on students with health impairments are typically taught as part of special education coursework; however, many of these students are served in the general education setting, and many general education teachers do not receive this type of instruction (Murphy et al., 2018). Consultations, workshops, and computer-based training have also been used to support community school personnel. Inservice teachers have reported feeling significantly more prepared and knowledgeable after completing computer-based training and have described such training as practical and helpful. Indeed, pre- and posttest comparisons of this type of training show these programs can improve teachers’ knowledge regarding pediatric cancer and how to support patients in the classroom (Brown et al., 2011; Dubowy et al., 2006).

Without specific training on how to best support students with cancer, teachers feel unprepared. They report that if they had specialized training or education on supporting students during school reentry, they would be more consistent, patient, and supportive of patients re-entering their classrooms. Instead, they feel unsure about realistic expectations and worry about the perceptions of classmates (Thompson et al., 2015). In a survey conducted by Nabors and colleagues (2008), a minority of teachers reported being very well informed regarding medical conditions, including cancer, in their classrooms, and teachers generally did not have high confidence in their ability to meet the social and academic needs of children with medical conditions. Overall, school personnel have positive attitudes toward students with medical conditions in their classrooms, but disease-specific concerns certainly exist (Olson et al., 2004). Specifically, teachers worry about a medical emergency, the disease’s impact on academic performance, and the extra demand for teacher attention the student may require.

## The Present Study

The aims of this quality improvement project were to evaluate community school personnel’s perceptions of their preparedness to work with patients and survivors of cancer and to standardize an approach to providing school reentry support for those community school personnel. First, community school personnel were surveyed regarding their perceptions of their preparedness to meet the needs of pediatric cancer patients and survivors who have returned to their classroom. In turn, educational guides were created with information and resources to fill the gaps in preparedness.

Historically, the authors have provided school reentry supports to patients, families, peers, and community school personnel. Our pediatric hospital primarily serves patients with hematological and oncologic diagnoses who come from all over the United States for treatment. Until now, no standardized intervention has been provided systematically within the institution, and the current supports and services had not been evaluated with input from community school personnel.

## Method

### Design

An online survey was created to assess the needs of community school personnel with regard to their preparedness to support returning cancer patients to their classrooms. Surveys were sent to school staff who had a patient return to their classroom during or after cancer treatment in the 2018–2019 school year. The five-question survey was designed to be completed quickly while allowing respondents the opportunity to ask for follow-up or additional clarification by leaving their contact information. The survey link and rationale for the project were sent via email to participants in the spring of 2020. The survey included the following free-response questions:

- What were your most significant concerns when the patient returned to the classroom?
- What information did you receive about the patient before their return to the classroom?
- What information would have been most helpful to know when the patient returned to the classroom?

School personnel were also asked to rate the statement “I understood how the student’s diagnosis and treatment could impact their school performance” using a 5-point Likert scale ranging from “did not understand” to “completely understood.” The final, fifth, question was open-ended, asking the respondent to share additional information, as desired.

Open-ended survey responses were compiled and analyzed using theoretical thematic analysis as outlined by Braun and Clarke (2006). Steps included reviewing responses, generating code, and defining themes. Three of the authors independently reviewed survey responses and conducted initial coding for each question. During the subsequent phase, a thematic map was created for each question to conceptualize the response patterns. Themes were identified from repeated key features of the initial codes. Main themes and subthemes were devised. Collated extracts were reviewed under each subtheme for continuity and coherence. Subthemes were reviewed and refined, and any disagreements were resolved by consensus discussion. Individual themes were reviewed in relation to each survey question to ensure accurate representation of the entirety of survey responses. Finally, themes were defined and named.

### Participants

Community school personnel contact information was retrieved from the school records of patients receiving instructional services through the institution’s hospital school program while undergoing cancer-directed therapy during the 2018–2019 school year. Sixty students were selected using a random number generator, and their primary school contact was recorded. Some students, especially those in middle or high school with multiple teachers, had multiple school contacts. In cases where a primary school contact could not be established, the survey link was sent to multiple community school personnel. A total of 66 emails were sent out; of these, 10 were returned as the recipient address was no longer valid.

Twenty community school personnel completed the survey, for a 36% response rate. School personnel positions are reported in ***Table 1***. Grade-level positions (K-6, 7-12) were split to mirror those of the hospital school program. Other respondents included a prekindergarten/kindergarten special education teacher and two 6^th^- to 8^th^-grade classroom teachers. Half of the respondents were teachers working directly with the student; the other half was evenly split between guidance counselors and administrators.

**Table 1:** School Personnel Positions.


POSITION	NUMBER OF RESPONSES

**K-6 Classroom Teacher**	*n* = 4

**7-12 Classroom Teacher**	*n* = 2

**Homebound Teacher**	*n* = 1

**Guidance Counselor**	*n* = 5

**Administrator**	*n* = 5

**Other**	*n* = 3

**Total**	*N* = 20


*Note*: Other respondents included two 6^th^– to 8^th^-grade classroom teachers and one prekindergarten/kindergarten special education teacher.

## Results

In response to the statement “I understood how the student’s diagnosis and treatment could impact their school performance,” using a 5-point Likert scale ranging from “did not understand” to “completely understood,” 90% (*n* = 18) of respondents reported they “completely understood;” the remaining 10% (*n* = 2) fell between “completely understood” and “somewhat understood.” Although all the respondents reported high levels of understanding the implications of diagnosis and treatment, a variety of concerns were reported on the free response items.

### Significant Concerns at School Reentry

#### Student Readiness

**Figure 1:**
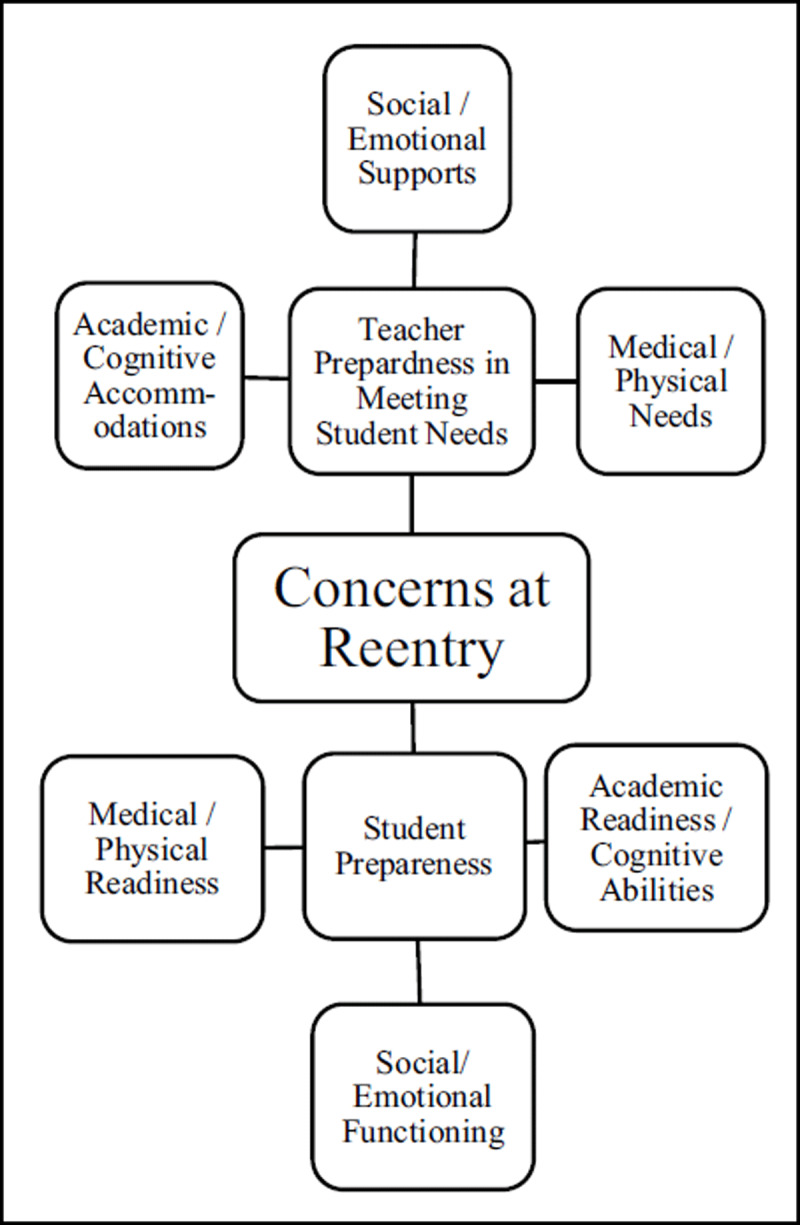
School Personnel’s Most Significant Concerns at School Reentry.

A major theme that emerged from analyzing school personnel’s most significant concerns was the student’s readiness for reentry. Subthemes included the student’s academic/cognitive readiness, social-emotional functioning, and medical/physical abilities, as noted in ***Figure 1***. School personnel (*n* = 9) were specifically concerned about academics, for example, the amount of work the student completed while they were away from the classroom and the potential for significant learning gaps compared to their peers. In addition, questions regarding the cognitive impact of treatment and the patient’s level of functioning were raised. One respondent noted their student needed one-on-one tutoring when they returned to the classroom, and another stated that the student “was not functioning at a level even close to previous performance.”

School personnel (*n* = 12) were also concerned about the social-emotional impact of treatment and returning to the classroom and wanted to be sure the student was emotionally prepared to be with their peers and acclimate back to the learning environment. One respondent wanted to be sure the patient was “adjusting to being back and dealing with such a serious and different reality than most of her peers.”

Lastly, school personnel wanted reassurance that the patient was medically cleared and had the physical stamina to return, with eight respondents specifically mentioning medical or physical concerns.

#### School Personnel Preparedness

Another major theme that emerged from the analysis of school personnel’s most significant concerns was the school team’s preparedness for the student’s return. Subthemes included the school team’s ability to meet the student’s academic, social-emotional, and physical needs when they returned to the classroom (see ***Figure 1***). School personnel (*n* = 3) worried about how they could academically support the student during the transition back to the classroom. One respondent noted, “It was hard because academically she is way behind and almost not capable of some of the work. Socially it is important for her to be with her peers.” Some school teams (*n* = 5) wanted to ensure they were providing appropriate accommodations for the patient to physically access the classroom and/or school building. Concerns regarding emotionally supporting the student included teachers trying to “ease anxiety” and “trying to get them to relax.” A school staff member noted they hoped the student would “feel welcomed back,” and another hoped this transition would be “a very smooth, low stress reentry.” Six respondents mentioned a general lack of preparedness and asked questions about returning students such as, “What could she handle?” and “What should I do to help her learn the most/best way possible?”

### Information Received by School Personnel

Information received by school personnel included details about academic progress or cognitive concerns, social-emotional functioning, and physical and medical considerations. The majority of school personnel (*n* = 17) responded receiving some type of information from the student’s family or hospital school program staff. Academic information might include some or all the following: the results of classroom-based assessments, grade reports, a progress summary by the hospital teacher, information on gaps in learning, suggested accommodations, and work samples collected during treatment.

Half of the respondents specifically mentioned receiving academic information or being in contact with a hospital teacher during the patient’s treatment. Two respondents reported receiving information on the patient’s social-emotional adjustment. Medical information received might have included details on diagnosis, updates on the patient’s condition, and prognosis.

Three of the 20 respondents reported receiving no information at reentry. One respondent commented that they received information from their school’s counseling department but wished they knew more. Another respondent reported that the patient finished treatment at the end of the school year, so they did not receive any information due to school being closed for the summer.

### Information Wanted by School Personnel

School personnel reported wanting information regarding the patient’s academic progress and cognitive abilities, social-emotional functioning, and medical needs. Seven of the 20 respondents specifically noted a need to be informed regarding academic progress and cognitive abilities, including topics covered or skills learned during treatment and cognitive evaluation or achievement test results. The need for insights on social concerns and guidance for how to provide “emotional accommodations” was also mentioned. School personnel (*n* = 6) wanted a wide range of medical information, including symptoms to watch for, follow-up appointment schedule, recovery process, medical history, stamina concerns, and physical abilities. Three of the respondents noted they did not want any additional information other than what they had received from the hospital teacher.

## Summary

This study’s results support the need for systematic support for community school personnel working with pediatric cancer patients and survivors. Using a Likert-scale rating, most respondents reported that they understood how treatment and diagnosis may impact school functioning. However, the contrast between this rating and the significant concerns and gaps in preparedness described in the free-response questions highlights the need to provide tailored guidance and specific information to school personnel. That is, while they reported a generalized understanding of treatment, diagnosis, and school impact, most school personnel surveyed still wanted additional information to aid in their understanding.

School personnel often struggled with deciding “what was most important” when trying to balance the student’s academic, social, and physical needs. Respondents had many concerns regarding their preparedness and ability to compensate for the student’s needs. This sentiment was true for teachers working directly with the student in the classroom as well as other school personnel, such as guidance counselors or administrators who may not have day-to-day interactions with the patient. Regardless of position within the school building or district, survey responses echoed similar needs and concerns.

### Standardizing Reentry Supports

In addressing the second aim for this project, feedback from community school personnel was used to develop standardized informational teacher guides. During the guide revision process, the authors recognized that the needs and recommendations for each patient were dependent on their cancer diagnosis, treatment received, and phase of treatment. Therefore, diagnosis-specific guides were created for two time points: at diagnosis (*Pediatric Cancer in the Schools: A Guide for Working With Students Receiving Cancer Treatment*) and at school reentry (*Pediatric Cancer in the Schools: A Guide for Working With Students Returning to the Classroom*). (See supplementary files for guides.)

**Figure 2:**
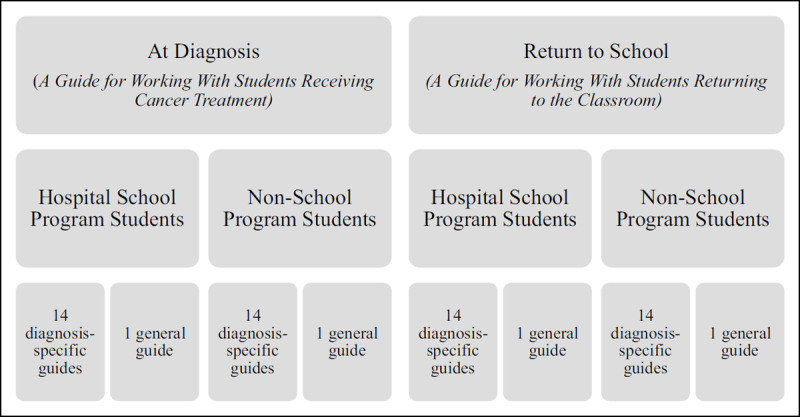
Categories of Teacher Guides Provided to Community School Personnel. *Note*: Hospital school program students are patients who receive instruction through the institution’s hospital school program. Non-school program students are patients who receive instruction through their community school.

Some patients can attend their community school while receiving treatment at our institution, while nearly 85% of patients receive instructional services through the hospital’s school program, so guides were also tailored for these two patient populations. Guides were developed for the 14 most common oncological diagnoses treated at the hospital, including specific information about diagnosis, treatment, and side effects. One guide was also developed with general information about childhood cancer. In total, 60 unique guides were created (see ***Figure 2*** for categorization of the guides).

While each guide is unique, ***Table 2*** provides a general outline of the content. Content was written specifically to include the information school personnel wanted to know at school reentry based on survey feedback; that is, survey results directly informed guide content. Specific content for diagnoses, time points in the treatment trajectory, and patient populations provides school personnel with more tailored guidance and information based on their student’s reentry. For example, *Pediatric Cancer in the Schools: A Guide for Working With Students Receiving Cancer Treatment* includes information about what school may be like during treatment, academic support and educational plans, social-emotional support, medical and physical considerations, specific information about treatment, side effects, and recommendations for the classroom, along with additional resources for schools. Much of the same information is adapted in *Pediatric Cancer in the Schools: A Guide for Working With Students Returning to the Classroom*, which is provided to schools at reentry. Information about long-term side effects, details about the role of the school advocacy coordinator/school liaison after treatment, a return-to-school checklist, and more resources for schools are also included. The guides were designed for use in any school setting in the United States. All the content will not necessarily be relevant in every school setting or for every patient’s reentry; however, the guides are applicable across most circumstances.

**Table 2:** Outline of Teacher Guides.


A GUIDE FOR WORKING WITH STUDENTS RECEIVING CANCER TREATMENT

Introduction to Childhood Cancer General statistics Types of treatment Prognosis factors

School During Treatment Importance of maintaining normalcy Logistics of hospital-school programming or consultation process Logistics of homebound instruction Differences in decision-making timelines between hospitals and schools

Academic Support and Educational Plans Educational impact of disease and treatment Potential need for IEP/504 Plan

Social-Emotional Support Bullying issues Supporting peers and social relationships Acknowledgment of fear/uncertainty from school staff

Medical and Physical Considerations Weakened immune system/fatigue Managing treatment schedule and academic instruction Excusing medically related absences

Treatment Details (specific to the student) Specific therapies Duration of treatment plan Prognosis

Side Effects (specific to the student’s diagnosis and treatment) Possible school accommodations and supports

Resources for Schools

**A GUIDE FOR WORKING WITH STUDENTS RETURNING TO THE CLASSROOM**

Introduction to Childhood Cancer General statistics Types of treatment Prognosis factors

Returning to School at Home/School After Treatment Importance of maintaining normalcy Support available from hospital staff/consultation process

Academic Support and Educational Plans Long-term cognitive impact of cancer Potential need for an IEP or 504 Plan now or as school demands increase Differences in decision-making timelines between schools and hospitals

Social-Emotional Support Mixed emotions from student/family Possibility of survivor’s guilt Acknowledgment of fear/uncertainty from school staff Importance of open and regular communication

Medical and Physical Considerations Weakened immune system/fatigue Excusing medically related absences

Treatment Details (specific to the student) Specific therapies Prognosis Necessity of follow-up appointments

Side Effects (specific to the student’s diagnosis and treatment) Possible school accommodations and supports

Return-to-School Checklist Schedule a meeting with the family to discuss need for accommodations Determine attendance status (full-time, part-time, homebound) Discuss school reentry presentation, if appropriate Develop/update IEP, 504 Plan, or health care plan Assign a “point person” to check in with student at regular intervals

Resources for Schools


In order to ensure that systematic support is provided, our program’s standard procedures were updated to include sharing these guides. Guardians sign a release of information to include permission to share information about diagnosis, treatment, and recommendations for the classroom with their community school as part of their initial school consultation. Hospital school teachers electronically share *A Guide for Working With Students Receiving Cancer Treatment* as part of their initial school contact, typically within two weeks of diagnosis. For patients not receiving instructional services, the principal of hospital school program establishes contact with the school and provides the guide within that same timeframe. The guide is subsequently shared with classroom teachers, guidance counselors, or whomever is serving as the primary point person at the community school. As part of the school reentry planning process, hospital school teachers send *A Guide for Working With Students Returning to the Classroom* to the community school point person. This guide is typically emailed along with recommendations for accommodations, grade reports, and other pertinent school information after the patient’s last school session. For patients not receiving instructional services, communication with the community school is transferred from the principal to a school advocacy coordinator/school liaison, who sends the guide during the last few weeks of treatment. Standardizing the procedures for sharing the guides ensures community school personnel are prepared with the right information at the right time.

## Conclusion

Specifically stated in *Pediatric Blood & Cancer*’s psychosocial standards of care is the need to provide information to school personnel about the patient’s diagnosis, treatment, impact on learning, and recommendations on how to support the student in the classroom (Thompson et al., 2015). This same recommendation has been issued by other professional organizations in the field, including APHOES and SIOP. The survey results from this study concur: The challenges associated with the process of students reentering school after diagnosis and treatment can be mitigated by providing systematic reentry supports to community school personnel. While well-intentioned, community school personnel often lack the knowledge needed to meet the student’s academic, social-emotional, and physical needs upon returning to the classroom. They report a lack of preparedness, and the need for strategic support is evident.

To meet this need, the informational guides were developed using survey data from school personnel. The gaps in knowledge reported by school personnel reflect the essential elements recommended to meet the academic continuity and school reentry support psychosocial standard of care (Wiener et al., 2020). This reflection further highlights the importance of providing comprehensive education and guidance to community school personnel. While these guides cannot take the place of well-formed hospital-school-family partnerships, the outline for these guides can be adapted and used across multiple settings and programs to meet the needs of diverse patient populations and varied educational/school programs afforded to children’s hospitals. According to Thompson and colleagues (2015), the most significant barrier to providing standardized school reentry supports is the cost of programming and personnel. To counter this barrier, our guides can quickly and easily be attached to an email, so there is no cost for printing or materials and minimal time is spent on the part of hospital staff. Providing these guides to community school personnel can be integrated into a standardized approach to providing support at school reentry.

### Limitations of the Study

It is significant to note that the survey results were analyzed through the lens of hospital school personnel. Hospital-school-family communication is highly valued in the field, so every author had the opportunity to understand the perspective of community school personnel prior to survey completion. Preconceived bias about the needs of community school personnel and experience communicating with community school personnel may have influenced the thematic analysis; however, we believe the final themes and subthemes reflect the true message of community school personnel.

The low response rate from community school personnel may be due to several factors. First, recipients of the survey may have had limited direct interaction with the student upon their return home and, therefore, felt ill equipped to answer the survey questions. Second, survey questions were not piloted, so we were unable to discern if respondents truly understood the questions and/or if they understood the questions similarly. Third, school personnel surveyed worked with students who had received instructional services in the hospital school program during treatment. If a student did not receive instruction from the hospital school program, their community school likely did not receive any information regarding the impact of treatment or recommendations for accommodations at school reentry. Surveying this group may have yielded vastly different responses.

Finally, it is important to remember that the current survey results were obtained, and guidelines were created, in the United States. The guides would need to be carefully reviewed and evaluated before being used in school settings in other cultural contexts to ensure social and cultural appropriateness. For example, in the United States, 504 Plans are legislated under Section 504 of the Rehabilitation Act of 1973, which is a civil rights statute that requires school districts to provide eligible students with disabilities appropriate educational supports and services to the same extent as students without disabilities, whereas Individualized Education Programs (IEPs) are legislated under the Individuals with Disabilities Act, a law that governs how early intervention, special education, and related services are provided to students with disabilities (U.S. Department of Education, 2020). Academic supports and services are often formalized under a wide variety of programs and names, and the rights and responsibilities of school systems vary greatly; however, the guides can be adapted to suit educational practices across the globe.

### Implications for Further Study

The guides were developed as part of a quality improvement (QI) initiative to standardize information and supports shared from our hospital school program to community school personnel with the goal of increasing their preparedness to manage the unique academic, social-emotional, physical, and medical needs of pediatric cancer patients and survivors in the classroom. The Agency for Healthcare Research and Quality (2013) notes, “Although QI models vary in approach and methods, a basic underlying principle is that QI is a continuous activity” (3). Evaluation of outcomes related to the application of the guides from the perspective of multiple stakeholders (e.g., community school personnel, students, parents) will be necessary for continuous improvement. Outcome research will be required to measure the efficacy of the guides as an intervention to increase preparedness. To evaluate the application of the informational guides, it would be beneficial to survey personnel from schools that have received the guides. It will also be important to further explore the needs of school personnel based on specific demographics such as the returning student’s diagnosis and treatment, location of the school district (e.g., urban, suburban, rural), and setting (e.g., public, private). In addition, school personnel should be surveyed at multiple timepoints to highlight any gaps in knowledge and ensure that essential information is provided at diagnosis and at school reentry. It may also be informative to extend the time between school reentry and dissemination of the survey to determine how well the information follows students as they matriculate through their education. Additional investigation into the best methods for disseminating this information to the various personnel within a patient’s school will strengthen our ability to ensure that all members of a child’s educational team have access. Understanding how information is shared among school teams would be helpful in determining the utility of our intervention. Ultimately, future investigation should seek to measure the impact of the guides on school-related survivor outcomes (e.g., academic achievement, social functioning). This may be achieved using qualitative interviews and surveys with survivors and parents, as well as more objective proxies such as grade-point averages and graduation rates.

In the future, providing this content as an interactive training module rather than a written guide may be more effective in meeting the needs of community school personnel. Computer-based training programs have proven effective in increasing school personnel’s understanding of cancer, and school personnel have indicated an interest in using this type of platform to meet their continuing education needs (Brown et al., 2011; Dubowy et al., 2006). A comparison study would be useful in determining the utility and benefit between multiple learning platforms. Finally, further study will help us to reassess and determine if we need to change methods of communication or redevelop the QI initiative as part of the continuous improvement cycle.

## Additional Files

The additional files for this article can be found as follows:

10.5334/cie.36.s1 Appendix A. *Pediatric Cancer in the Schools: A Guide for Working With Students Receiving Cancer Treatment.* General guide provided to community school personnel at diagnosis.

10.5334/cie.36.s2 Appendix B. *Pediatric Cancer in the Schools: A Guide for Working With Students Returning to the Classroom.* General guide provided to community school personnel at school reentry.

Additional guides are available for specific oncology diagnoses by contacting the first author.
