# Supplementary material for: Support for Community School Personnel Working with Pediatric Cancer Patients: A Quality Improvement Initiative
Source: Contin Educ. 2022 Jan 20;3(1):1–12. doi: 10.5334/cie.36 (PMC11104404; doi:10.5334/cie.36)
Supplement: Appendix A. — Pediatric Cancer in the Schools: A Guide for Working With Students Receiving Cancer Treatment. General guide provided to community school personnel at diagnosis. [file cie-3-1-36-s1.pdf]

Pediatric Cancer in the Schools: A Guide for Working with Students Receiving Cancer Treatment

St. Jude Children's Research Hospital
Finding cures. Saving children.
ALSAC • DANNY THOMAS, FOUNDER

St. Jude School Program

Introduction to Childhood Cancer

Childhood cancers are rare, complex diseases. Caring for children is more than just treating the cancer. Promoting mental and physical health is important for both cure and quality of life.

School gives patients a chance to keep a sense of identity and normalcy.

Cancer does not exclusively occur in adults; it also occurs in infants, children, and teens. About 15,000 children are diagnosed with pediatric cancer each year in the United States. Treatment for childhood cancer might include surgery, chemotherapy, radiation therapy, immunotherapy, and targeted therapy. The treatment or combination of treatments used depend on several factors such as the type of cancer, the size and location of the tumor, and the age of the patient.

For many types of childhood cancer, treatment improvements have increased survival rates, which now average 80% or higher overall in the United States. However, the prognosis remains poor for some types of cancer. Scientists are learning more about the genetic causes of cancer and specific features of cancer cells. These discoveries will continue to improve the diagnosis and treatment of pediatric cancer.

School During Treatment

School is a normal part of childhood and adolescence. For students undergoing treatment, school can offer a familiar and reassuring routine, as well as a feeling of being in step with the outside world. Our school staff strive to make St. Jude patients feel like students first – in the rest of this packet, the St. Jude patient will be referred to as 'the student' as much as possible.

The St. Jude School Program is accredited as a Special Purpose School by Cognia. When a new patient is admitted to St. Jude, school staff meet with the patient and family to determine if they need assistance with school services. This assistance may include consulting with your school team to set up homebound services or to initiate a 504 Plan, IEP, or health plan. During this first meeting, Release of Information forms are signed so that St. Jude school staff can communicate with you. Consultation is an interactive, collaborative, problem-solving process which empowers parents and school personnel to best meet the educational needs of the student. Our goal in consultation is to improve parents' and school professionals' understanding of the student's unique needs in order to provide services that ensure academic success.

Hospitals and schools operate very differently. At St. Jude, families get used to problems being assessed and treatment being prescribed quickly. In schools, data is collected over longer periods of time in order to define and evaluate a student's needs. Families may find it difficult to move from one setting to the other and may feel frustration in the differences in timing. This time may also be the family's first experience with special education. The student and family will benefit from clear explanations and open conversations

Academic Support & Educational Plans

The educational impact of cancer is immediate and can be long-lasting. Students may struggle in school in ways they didn't before their diagnosis and treatment. Some treatments can affect memory, attention, processing, and organization. St. Jude patients are generally well-served with classroom accommodations under a 504 Plan. Some patients, particularly those who experience long-lasting impacts, may require more individualized, intensive services and supports under an IEP. Because of diagnosis and the impact of treatment, our patients can qualify for an IEP under "Other Health Impairment." If the student has been served under an IEP or 504 Plan prior to diagnosis, St. Jude school program staff can provide additional recommendations, if needed.

If the student is enrolled in online/virtual learning, allow for flexible sign-on times and durations. Due to changes in medical appointments and side effects of treatment, the student may benefit most from an asynchronous learning plan.

Social Emotional Support

The student may experience social emotional effects of their medical condition, changes in body image, loss of control, and social isolation. Parents may feel the need to protect the patient and may be overwhelmed by medical demands. School staff may experience fear due to lack of medical knowledge or a personal experience with cancer. Staff may be uncertain about their role in supporting the student or managing side effects in the classroom.

The student is going through experiences that are rare for people the same age. Friends may have disappeared during treatment, and the student may withdraw from social relationships. Some studies have shown that childhood cancer patients report approximately three times as much bullying as their healthy peers. This may be the case even if they are not in the classroom.

Encourage communication between the student and their peers. For peers, using social media may help them to see and understand what is happening during treatment. They can see changes in appearance as they happen and be able to ask questions and discuss these changes openly. Include the student in special events at school, such as holiday parties or Prom. **Ask the student and their family about their preferences on how much they want to be included and what information they want shared with their classmates; these preferences may change throughout treatment, so check in at regular intervals.**

Medical & Physical Considerations

Attending school all day, five days a week may not always be possible. Many students experience fatigue and weakness and cannot physically make it through a full day of school. Some students may have a weakened immune system and are not able to be in large groups of people, or others may have physical challenges that affect their mobility. It is important for school personnel and classmates to continue to practice good hygiene (handwashing) to help prevent the spread of germs. Inform families of any sicknesses or illness outbreaks at school, so they can make informed decisions regarding attendance for the student. **The student's specific needs should be addressed before returning to the classroom and re-evaluated often.**

Keep high but reasonable expectations for the student and their school work. Treatment schedules and side effects often mean academic instruction or homebound meeting times must be rescheduled. Flexible due dates, modified assignments, and the ability to work ahead (if possible) allow students to work when they feel well or when they are away from the hospital. Excuse all medically related absences, and plan for the student to receive their work prior to their absence.

Treatment

Treatment of pediatric cancer depends on the diagnosis, stage of the disease, and location of the disease, among other factors. Learn more about potential therapies in your student's treatment plan here.

Side Effects

Doctors plan treatments to limit side effects as much as possible while still treating cancer. Some students may have mild side effects while others may have more severe problems. Accommodations at school may be necessary for the student to succeed in the classroom. Some students may also require related special education services. Common side effects of cancer and its treatment are listed below with some potential school supports that might be offered. Please note, this list is not meant to be exhaustive.

Side Effect		Possible School Accommodations
**Low Absolute Neutrophil Count (ANC) and Neutropenia**	The ANC is an estimate of the body's ability to fight infections, especially bacterial infections. Patients may need to avoid public places such as schools or wear a face mask to lower the risk of infection.	• Encourage hand washing & good hygiene<br>• Tell parents about illness in the classroom
**Cognitive Side Effects**<br><br>**"Chemo Brain"**	Patients and families may notice changes in thinking, attention, or memory. Patients may also have difficulties with executive functions - paying attention, organizing, using working memory, or recalling information. They may need help self-monitoring or controlling their impulses. Some changes are temporary, while others may be long lasting.	• Abbreviated assignments<br>• Assignment notebook<br>• Copies of notes<br>• Graphic organizers<br>• Oral testing / read aloud<br>• Posted daily schedules<br>• Preferential seating<br>• Quiet area for testing<br>• Tutoring
**Fatigue**	Pediatric cancer patients face numerous potential causes of physical and mental fatigue. Fatigue may impact attention, processing speed, decision making and ultimately learning.	• Abbreviated school days<br>• Extended time<br>• Physical activity as tolerated<br>• Use of elevator
**Hair Loss**	Hair loss (alopecia) can be one of the most upsetting side effects of treatment. Hair loss is a visible reminder of being sick. For patients trying to be "normal," this can have a big impact on well-being and quality of life.	• Allow hats/scarves
**Hearing Loss**	Hearing loss can affect speech, social relationships, learning, and academic achievement. Symptoms may include difficulty paying attention, trouble following directions, a drop in grades, or balance problems.	• Audio amplification systems<br>• Copies of notes<br>• Preferential seating
**Nausea and Vomiting**	Symptoms can vary from mild to severe and may occur before, during, or after treatment. Patients and families often report that nausea is one of the side effects that bothers them most.	• Access to nurse<br>• Individual Health Plan<br>• Breaks as needed
**Changes in Vision**	Eye problems may include blurred vision, double vision, drooping eyelids, uncontrolled eye movements, or strabismus.	• Copies of notes<br>• Oral testing/read aloud<br>• Preferential seating

Resources for Schools

Staying Connected: Facilitating the Learning Experience During & After Cancer Treatment
This free 5.5 hour continuing education (CE) program from the Leukemia & Lymphoma Society is designed to be completed at a time that is convenient to you – following registration, you will have up to 16 weeks to complete the program in its entirety and receive CE credit or up to 3 weeks for the non-accredited track.
https://www.lls.org/professional-education-webcasts/staying-connected-facilitating-learning-experience-during-after

Cure4Kids For Teachers
This free St. Jude program aims to support K-12 educators by offering professional development opportunities that provide them with tools for teaching the basic science of cancer formation, treatment, and prevention. Look under the Resources tab for lesson plans and worksheets.
https://www.cure4kids.org/teachers

Educating the Child with Cancer
Written by top researchers in the field, and balanced with parents' personal experiences, this resource focuses on educational issues for children treated for cancer.
https://www.tfaforms.com/4714141

Together
Powered by St. Jude, this site offers dependable information and a community of support for anyone facing childhood cancer.
https://together.stjude.org/

St. Jude School Program
This site has additional information about the St. Jude School Program and how we can work with your school team to ensure each student's academic needs are met while they are away from the classroom.
https://www.stjude.org/treatment/services/clinics-and-services/school-program.html

For more information about treatments, side effects, and prognoses of different types of pediatric cancers, follow this link.

More information about school supports and accommodations for students with cancer can be found here.

Information in this packet is adapted from www.stjude.org and www.together.stjude.org
