# Supplementary material for: Support for Community School Personnel Working with Pediatric Cancer Patients: A Quality Improvement Initiative
Source: Contin Educ. 2022 Jan 20;3(1):1–12. doi: 10.5334/cie.36 (PMC11104404; doi:10.5334/cie.36)
Supplement: Appendix B. — Pediatric Cancer in the Schools: A Guide for Working With Students Returning to the Classroom. General guide provided to community school personnel at school reentry. [file cie-3-1-36-s2.pdf]

Pediatric Cancer in the Schools: A Guide for Working with Students Returning to the Classroom

St. Jude Children's Research Hospital
Finding cures. Saving children.
ALSAC • DANNY THOMAS, FOUNDER

St. Jude School Program

General

Introduction to Childhood Cancer

Childhood cancers are rare, complex diseases. Caring for children is more than just treating the cancer. Promoting mental and physical health is important for both cure and quality of life.

The return to school at home is the student's hope for the future. It promotes learning, normal psychosocial development, and a sense of purpose.

Cancer does not exclusively occur in adults; it also occurs in infants, children, and teens. About 15,000 children are diagnosed with pediatric cancer each year in the United States. Treatment for childhood cancer might include surgery, chemotherapy, radiation therapy, immunotherapy, and targeted therapy. The treatment or combination of treatments used depend on several factors such as the type of cancer, the size and location of the tumor, and the age of the child.

For many types of childhood cancer, treatment improvements have increased survival rates, which now average 80% or higher overall in the United States. However, the prognosis remains poor for some types of cancer. Scientists are learning more about the genetic causes of cancer and specific features of cancer cells. These discoveries will continue to improve the diagnosis and treatment of pediatric cancer.

School After Treatment

School is a normal part of childhood. School gives patients a chance to keep a sense of identity and normalcy. As such, patients are typically encouraged to return to school as soon as medically appropriate. When they are with us, our school staff strive to make St. Jude patients feel like students first – in the rest of this packet, the St. Jude patient will be referred to as 'the student' as much as possible.

The educational impact of cancer does not stop at the completion of therapy or remission of disease. As treatment and cure rates continue to improve, more and more childhood cancer survivors live with long-term effects of disease and treatment, such as impairments to learning and thinking. St. Jude school advocacy coordinators can consult with your school team. Consultation is an interactive, collaborative, problem-solving process which empowers parents and school personnel to best meet the educational needs of the student. Our goal in consultation is to improve parents' and school professionals' understanding of the student's unique needs in order to provide services that ensure academic success. After a plan has been implemented, the school advocacy coordinator will continue to remain available for any additional educational needs or concerns.

Academic Support & Educational Plans

The educational impact of cancer is immediate and can be long-lasting. St. Jude patients are generally well-served with classroom accommodations under a 504 Plan. Some patients may require more intensive services and supports under an IEP, such as related rehabilitation services. Because of diagnosis and the impact of treatment, our patients can qualify for an IEP under “Other Health Impairment.” If the student was served under an IEP or 504 Plan prior to treatment, the team should meet to make revisions if necessary. The patient may also need a health care plan. Closely tracking student progress is important as our patients often experience “late effects” and need support later in their academic careers, as they move out from their treatment and as school demands increase.

Hospitals and schools operate very differently. At St. Jude, families get used to problems being assessed and treatment being prescribed quickly. In schools, data is collected over longer periods of time in order to define and evaluate a student’s needs. **Families may find it difficult to move from one setting to the other and may feel frustration in the differences in timing.** It may be the family’s first experience with special education. It may also be the first time the family has been able to look past being cured and think about the impact of treatment.

Social Emotional Support

Patients and families often have mixed emotions when they finish treatment. Patients may experience separation anxiety (from their parents or St. Jude), effects of the medical condition, changes in body image, loss of control, and social isolation. Parents may feel the need to protect the patient and may be overwhelmed by medical demands. School staff may experience fear due to lack of medical knowledge or a personal experience with cancer. Staff may be uncertain about their role in supporting the student or managing side effects in the classroom.

The student has gone through experiences that are rare for people the same age. While they may be excited to finish treatment, they are leaving friends behind at St. Jude. Our patients sometimes report feeling survivor’s guilt when they finish treatment or when a friend from St. Jude dies. Friends from home may have disappeared during treatment, and the student may withdraw from social relationships. Some studies have shown that childhood cancer patients report approximately three times as much bullying as their healthy peers. This may be the case even if they are not in the classroom.

As your student prepares to return to school, talk with the family and student about what they want their classmates to know and how they want their first day back to go. Oftentimes, they just want it to be an ordinary day. They may also want to plan some type of re-entry presentation to share information with peers.

Medical & Physical Considerations

Attending school all day, five days a week may not be possible at first. Many students experience fatigue and weakness and cannot physically make it through a full day of school. Some students may have a weakened immune system and are not able to be in large groups of people, or others may have physical challenges that affect their mobility. It is important for school personnel and classmates to continue to practice good hygiene (handwashing) to help prevent the spread of germs. Inform families of any sicknesses or illness outbreaks at school, so they can make informed decisions regarding attendance for the student. **The student’s specific needs should be addressed before returning to the classroom and re-evaluated often.** Keep high but reasonable expectations for the student and their school work. After finishing treatment, they will still have medical follow-up appointments. Excuse all medically related absences, and plan for the student to receive their work prior to their absence.

Treatment

Treatment of pediatric cancer depends on the diagnosis, stage of the disease, and location of the disease, among other factors. Learn more about potential therapies in your student's treatment plan here.

Side Effects

Doctors plan treatments to limit side effects as much as possible while still treating cancer. Some students may have mild side effects while others may have more severe problems. Accommodations at school may be necessary for the student to succeed in the classroom. Some students may also require related special education services. Common side effects of cancer and its treatment are listed below with some potential school supports that might be offered. Please note, this list is not meant to be exhaustive.

Side Effect		Possible School Accommodations
**Low Absolute Neutrophil Count (ANC) and Neutropenia**	The ANC is an estimate of the body's ability to fight infections, especially bacterial infections. Patients may need to avoid public places such as schools or wear a face mask to lower the risk of infection.	• Encourage hand washing & good hygiene<br>• Tell parents about illness in the classroom
**Cognitive Side Effects**<br><br>**"Chemo Brain"**	Patients and families may notice changes in thinking, attention, or memory. Patients may also have difficulties with executive functions - paying attention, organizing, using working memory, or recalling information. They may need help self-monitoring or controlling their impulses. Some changes are temporary, while others may be long lasting.	• Abbreviated assignments<br>• Assignment notebook<br>• Copies of notes<br>• Graphic organizers<br>• Oral testing / read aloud<br>• Posted daily schedules<br>• Preferential seating<br>• Quiet area for testing<br>• Tutoring
**Fatigue**	Pediatric cancer patients face numerous potential causes of physical and mental fatigue. Fatigue may impact attention, processing speed, decision making and ultimately learning.	• Abbreviated school days<br>• Extended time<br>• Physical activity as tolerated<br>• Use of elevator
**Hair Loss**	Hair loss (alopecia) can be one of the most upsetting side effects of treatment. Hair loss is a visible reminder of being sick. For patients trying to be "normal," this can have a big impact on well-being and quality of life.	• Allow hats/scarves
**Hearing Loss**	Hearing loss can affect speech, social relationships, learning, and academic achievement. Symptoms may include difficulty paying attention, trouble following directions, a drop in grades, or balance problems.	• Audio amplification systems<br>• Copies of notes<br>• Preferential seating
**Nausea and Vomiting**	Symptoms can vary from mild to severe and may occur before, during, or after treatment. Patients and families often report that nausea is one of the side effects that bothers them most.	• Access to nurse<br>• Individual Health Plan<br>• Breaks as needed
**Changes in Vision**	Eye problems may include blurred vision, double vision, drooping eyelids, uncontrolled eye movements, or strabismus.	• Copies of notes<br>• Oral testing/read aloud<br>• Preferential seating

Return to School Check List

This checklist may be useful in making sure returning to school after treatment is a smooth process.

- ☐ When notified that the student is returning to school, schedule a meeting with the family to discuss the need for accommodations or support in the classroom. If any paperwork needs to be completed for homebound or re-enrollment, provide it to the family at this time.
- ☐ Determine if the student will return to school full-time or start with half days. Often, students will need homebound services initially, and then return to school.
- ☐ Discuss whether a school re-entry presentation is appropriate. The student may have created something to share with their classmates while they were at St. Jude.
- ☐ Develop or update a 504 Plan, IEP, or health care plan to meet the student's needs.
- ☐ Assign a "point person" who will check in with the student to discuss any adjustment issues or concerns at a regular interval.

Resources for Schools

Staying Connected: Facilitating the Learning Experience During & After Cancer Treatment
This free 5.5 hour continuing education (CE) program from the Leukemia & Lymphoma Society is designed to be completed at a time that is convenient to you – following registration, you will have up to 16 weeks to complete the program in its entirety and receive CE credit or up to 3 weeks for the non-accredited track.
https://www.lls.org/professional-education-webcasts/staying-connected-facilitating-learning-experience-during-after

Cure4Kids For Teachers
This free St. Jude program aims to support K-12 educators by offering professional development opportunities that provide them with tools for teaching the basic science of cancer formation, treatment, and prevention. Look under the Resources tab for lesson plans and worksheets.
https://www.cure4kids.org/teachers

Educating the Child with Cancer
Written by top researchers in the field, and balanced with parents' personal experiences, this resource focuses on educational issues for children treated for cancer.
https://www.tfaforms.com/4714141

Together
Powered by St. Jude, this site offers dependable information and a community of support for anyone facing childhood cancer.
https://together.stjude.org/

St. Jude School Program
This site has additional information about the St. Jude School Program and how we can work with your school team to ensure each student's academic needs are met while they are away from the classroom.
https://www.stjude.org/treatment/services/clinics-and-services/school-program.html
